# The FACT-8D, a new cancer-specific utility algorithm based on the Functional Assessment of Cancer Therapies-General (FACT-G): a Canadian valuation study

**DOI:** 10.1186/s12955-022-02002-z

**Published:** 2022-06-16

**Authors:** Helen McTaggart-Cowan, Madeleine T. King, Richard Norman, Daniel S. J. Costa, A. Simon Pickard, Rosalie Viney, Stuart J. Peacock, the Canadian MAUCa Team, Kelvin Chan, Jeffery Hoch, Natasha Leighl, Nicole Mittmann, Dean Regier

**Affiliations:** 1 Cancer Control Research, BC Cancer, 675 West 10th Avenue, Vancouver, BC V5Z 1L3 Canada; 2 Faculty of Health Sciences, Simon Fraser University, Burnaby, BC Canada; 3 School of Psychology, University of Sydney, Sydney, Australia; 4 School of Public Health, Curtin University, Perth, Australia; 5 Sydney Medical School, University of Sydney, Sydney, Australia; 6 Pain Management Research Institute, Royal North Shore Hospital, Sydney, Australia; 7 Department of Pharmacy Systems, Outcomes, and Policy, University of Illinois at Chicago, Chicago, USA; 8 Centre for Health Economics Research and Evaluation, University of Technology, Sydney, Australia

**Keywords:** Canada, Cancer, Discrete choice experiment, FACT-G, health-related quality of life, utility

## Abstract

**Introduction:**

Utility instruments are used to assess patients’ health-related quality of life for cost-utility analysis (CUA). However, for cancer patients, the dimensions of generic utility instruments may not capture all the information relevant to the impact of cancer. Cancer-specific utilities provide a useful alternative. Under the auspices of the Multi-Attribute Utility in Cancer Consortium, a cancer-specific utility algorithm was derived from the FACT-G. The new FACT-8D contains eight dimensions: pain, fatigue, nausea, sleep, work, support from family/friends, sadness, and worry health will get worse. The aim of the study was to obtain a Canadian value set for the FACT-8D.

**Methods:**

A discrete choice experiment was administered to a Canadian general population online panel, quota sampled by age, sex, and province/territory of residence. Respondents provided responses to 16 choice sets. Each choice set consisted of two health states described by the FACT-8D dimensions plus an attribute representing survival duration. Sample weights were applied and the responses were analyzed using conditional logistic regression, parameterized to fit the quality-adjusted life year framework. The results were converted into utility weights by evaluating the marginal rate of substitution between each level of each FACT-8D dimension with respect to duration.

**Results:**

2228 individuals were recruited. The analysis dataset included n = 1582 individuals, who completed at least one choice set; of which, n = 1501 completed all choice sets. After constraining to ensure monotonicity in the utility function, the largest decrements were for the highest levels of pain (− 0.38), nausea (− 0.30), and problems doing work (− 0.23). The decrements of the remaining dimensions ranged from − 0.08 to − 0.18 for their highest levels. The utility of the worst possible health state was defined as − 0.65, considerably worse than dead.

**Conclusions:**

The largest impacts on utility included three generic dimensions (i.e., pain, support, and work) and nausea, a symptom caused by cancer (e.g., brain tumours, gastrointestinal tumours, malignant bowel obstruction) and by common treatments (e.g., chemotherapy, radiotherapy, opioid analgesics). This may make the FACT-8D more informative for CUA evaluating in many cancer contexts, an assertion that must now be tested empirically in head-to-head comparisons with generic utility measures.

**Supplementary Information:**

The online version contains supplementary material available at 10.1186/s12955-022-02002-z.

## Introduction

The cost of cancer therapies is constantly rising. Understanding the financial implications of these therapies on the healthcare system is important, but it is also important to understand the impact these therapies have on patients in order to assess their value. As such, there is a need for sensitive instruments that capture the different dimensions of health-related quality of life (HRQL) to reflect both the therapeutic benefit and the treatment burden experienced by patients with cancer. Ideally, responses to these instruments would provide a utility to inform cost-utility analysis (CUA), the preferred economic evaluation approach by many health technology assessment organizations [1–3]. Conventionally, due to their ease in administration, “off the shelf” generic multi-attribute utility instruments (MAUIs) (e.g., the EQ-5D [4, 5] or the Health Utility Index (HUI) [6, 7]) are used to generate utilities, which are then suitable for estimating QALYs. Utilities are measured on a scale, where one represents full health, 0 represents states deemed to be as bad as being dead, and negative values represent states deemed to be worse than being dead. However, by their nature, the dimensions of generic instruments cover ubiquitous aspects of health, but may be limited in their ability to capture specific impacts of particular conditions and treatments.

The Functional Assessment of Cancer Therapies-General (FACT-G) [8], a widely used cancer-specific patient-reported outcome (PRO) measure, is used often to generate HRQL endpoints in cancer clinical trials. It is a stand-alone HRQL profile measure, and is also included in the many other condition and treatment-specific questionnaires in the FACIT measurement suite [9]. The FACT-G enables patients to self-report the impact of their cancer and treatments, but the scores it yields cannot be used in CUA because they do not provide any information about the strength of individuals’ preferences for specific HRQL dimensions of the instrument or about the trade-off between HRQL and survival. Responses on the FACT-G serve as a description rather than a valuation of health states; as such, their outputs are not health state utilities.

One approach that enables FACT-G responses to be measured onto a utility scale is regressing utility scores from generic measures onto the FACT–G total score or its dimension subscores using datasets containing both measures. However, a review of cancer mapping studies indicated that these regressions generally show poor goodness-of-fit between the observed generic utilities and the mapped cancer-specific utilities [10]. Another approach is to derive a MAUI to generate utilities from data collected from the FACT-G. A disadvantage of this approach include the inability to compare results across programs designed for different conditions and the potential neglect of co-morbidities; however, cancer-specific MAUI ensure that the resulting utilities better reflect the impact of cancer when incorporated into a CUA. Further, having a cancer-specific MAUI based on the FACT-G would make better use of studies where the FACT-G has generated HRQL endpoints but generic MAUIs have not been included.

The Multi-Attribute Utility in Cancer (MAUCa) Consortium has derived a MAUI to generate utilities from data collected from the QLQ-C30 to produce the European Organization of Research and Treatment in Cancer (EORTC) QLU-C10D [11]. Since its development, a number of country-specific utility weights for the QLU-C10D have followed, including Canada [12–19]. Recently, the MAUCa Consortium derived the health state classification system from items of the FACT-G and the Australian value set for the new FACT-8D has now been produced [20]. However, there is a need to inform cancer priority setting and resource allocation decisions in other jurisdictions; as such, country-specific utility weights for the FACT-8D are needed. The aim of this current paper is to produce the FACT-8D value set for the Canadian general population as the Canadian Agency for Drugs and Technologies in Health (CADTH) recommends that the preferences of the Canadian general population should be the reference case [1].

## Methods

### FACT-8D health state classification system

The derivation of the FACT-8D health state classification system is described in detail elsewhere [20]. In brief, using existing FACT-G datasets: (1) confirmatory factor analysis verified the measurement model; (2) Rasch and psychometric analyses guided item selection; and (3) patient opinions informed item selection. The FACT-8D consists of eight dimensions, which map directly to nine items of the FACT-G (Table 1). The five levels of the FACT-G also described the FACT-8D dimensions: not at all, a little bit, somewhat, quite a bit, and very much. The name, FACT-8D, has been endorsed by FACIT.org [21]: ‘FACT’ indicates the origin of the instrument; and ‘8D’ indicates its eight dimensions.

**Table 1:** The FACT-8D health state classification system and mapping to FACT-G items

Dimension	DCE attribute wording	Level	Descriptor	FACT-G item scores
Pain	Pain	0	Not at all	GP4 = 0
		1	A little bit	GP4 = 1
		2	Somewhat	GP4 = 2
		3	Quite a bit	GP4 = 3
		4	Very much	GP4 = 4
Fatigue	Fatigue	0	Not at all	GP1 = 0
		1	A little bit	GP1 = 1
		2	Somewhat	GP1 = 2
		3	Quite a bit	GP1 = 3
		4	Very much	GP1 = 4
Nausea	Nausea	0	Not at all	GP2 = 0
		1	A little bit	GP2 = 1
		2	Somewhat	GP2 = 2
		3	Quite a bit	GP2 = 3
		4	Very much	GP2 = 4
Sleep^a^	Problems sleeping	0	Not at all	GF5 = 4
		1	A little bit	GF5 = 3
		2	Somewhat	GF5 = 2
		3	Quite a bit	GF5 = 1
		4	Very much	GF5 = 0
Work^a^	Problems doing work (including work at home)	0	Not at all	GF1 = 4
		1	A little bit	GF1 = 3
		2	Somewhat	GF1 = 2
		3	Quite a bit	GF1 = 1
		4	Very much	GF1 = 0
Support^a^	Problems with support from my family and/or friends	0	Not at all	GS2 OR GS3 = 4
		1	A little bit	GS2 OR GS3 = 3
		2	Somewhat	GS2 OR GS3 = 2
		3	Quite a bit	GS2 OR GS3 = 1
		4	Very much	GS2 OR GS3 = 0
Sadness	Sadness	0	Not at all	GE1 = 0
		1	A little bit	GE1 = 1
		2	Somewhat	GE1 = 2
		3	Quite a bit	GE1 = 3
		4	Very much	GE1 = 4
Worry my health will get worse	Worry my health will get worse	0	Not at all	GE6 = 0
		1	A little bit	GE6 = 1
		2	Somewhat	GE6 = 2
		3	Quite a bit	GE6 = 3
		4	Very much	GE6 = 4

^a^Reverse scoring is required for FACT-8D dimensions Sleep, Work and Support to map the DCE attribute wording correctly to corresponding FACT-G items GF5 (I am sleeping well), GF1 (I am able to work), GS2 (I get emotional support from my family) and GS3 (I get support from my friends)

The FACT-G consists of a collection of positively- and negatively-phrased items. For example, the Social/Family and Functional wellbeing dimensions consist of positively-phrased items such as “I feel close to my friends” and “I am able to work (include work at home)”, respectively; whereas, the Physical and Emotional wellbeing dimensions consist of negatively-phrased items such as “I have a lack of energy” and “I feel sad”, respectively. Initial work revealed inconsistency in the responses to the interim valuation task [20]: a large number of non-monotonic dimension levels and positive utilities. Members of the MAUCa Consortium speculated that the directional change in the phrasing of the FACT-8D dimensions contributed to this inconsistency. As a result, all FACT-8D dimensions that are positively phrased were revised, such that all dimensions were phrased in the negative direction. For example, “I am able to work (include work at home)”, “I am sleeping well”, and “I get support from my family and/or friends became “problems doing work (include work at home)”, “problems sleeping”, and “problems with support from my family and/or friends”, respectively.

### Discrete choice experiment

The FACT-8D health state classification system was valued in the Canadian setting using the discrete choice experiment (DCE) methodology developed for the Australian valuation. Details of the experimental design are reported elsewhere [20]. Briefly, the experimental design underpinning the DCE contained nine attributes: the eight FACT-8D dimensions and duration of survival. Because the descriptive system contains a large number of dimensions, the experimental design was specified such that only five attributes differed in each choice sets (e.g., four HRQL dimensions and duration), and these were highlighted in yellow to reduce the cognitive complexity of the task [22]. The DCE consisted of 16 choice sets, in which respondents had to choose between two health states—Situation A or Situation B—each with a specified duration of life years (four levels: 1, 2, 5, 10 years); which option seen as Situation A or Situation B was randomized within each choice set. The order of the dimensions was kept the same for each respondent, as dimension order has been shown to not systematically bias utility weights [23].

### Data collection

Sampling and survey administration for all country-specific valuations in the MAUCa Consortium were undertaken by SurveyEngine, a company that specializes in choice experiments [24]. For this study, respondents over 18 years of age from the Canadian general population were recruited from an online panel. Quota sampling ensured age, sex, and province/territory of residence aligned with the Canadian Census [25].

Respondents completed the following survey components: welcome/disclosure; sex and age (for screening and quota sampling); self-reported health (SF-36 general health question [26] and FACT-G general population version [FACT-GP] [27]; the DCE; respondent perception of the difficulty and clarity of the DCE choice task and strategies used; sociodemographic variables and self-reported mental health (Kessler-10) [28]. The study protocol was approved by the Research Ethics Board at BC Cancer and the University of British Columbia (H15-03293).

### Data analysis

#### Study sample

We used descriptive statistics to characterize the sample in terms of sociodemographic variables, perceived difficulty, and clarity of the DCE task and choice strategies. Chi-squared tests assessed the representativeness of the study sample in comparison to the Canadian general population [25].

#### Utility estimation

For variables that are non-representative by ≥ %2 for one or more response level, weights were derived using iterative proportional fitting, or raking, to improve the relationship between the survey sample and the population (ipfweight option in Stata). The sample weights were applied to the utility estimation.

As with the Canadian QLU-C10D valuation study [12], the DCE responses were analyzed using a functional form in which the FACT-8D dimension levels interacted with the duration variable [29]:

1 $$U_{isj} = \alpha TIME_{isj} + \beta X_{isj}^{{\prime }} TIME_{isj} + \varepsilon_{isj} ,$$

where *α* was the utility associated with a life year in full health, $X_{isj}^{{\prime }}$ was a set of dummy variables relating to the levels of the FACT-8D health state presented in option *j*, and *ε*_*isj*_ was a random error term distributed independently and identically normal. This approach has previously been used to estimate utilities from DCE data to ensure consistency with standard QALY model restrictions: (1) all health states have zero utility at dead state; and (2) the proportion of remaining life years that an individual is willing to give up for an improvement in health status does not depend on the absolute number of remaining life years (i.e., constant proportional time trade-off).

The modelling approach followed that used in the valuation studies conducted in Australia and the United States [14]. The DCE responses were analyzed using a conditional logit model (Model 1). A clustered sandwich estimator using the vce (cluster) option in STATA adjusted the standard errors to allow for intra-individual correlation as each respondent considered the 16 DCE choice sets. The impact of moving away from one level of each dimension is investigated through two-factor interaction terms using the continuous duration term and each dimension level (e.g., the effect of moving from level 1 to level 2 in the pain dimension is determined by using a pain level 2*duration interaction term).

After conducting the unweighted utility estimation (Model 1), derived sampling weights were then incorporated into the model (Model 2). A scatterplot compared the distribution of unweighted versus weighted coefficient estimates. If non-monotonic ordering was present between dimension levels in the conditional logit model, we conducted another conditional logit model after collapsing non-monotonic dimension levels (Model 3). A log-likelihood ratio test assessed the model fit between Model 2 and Model 3.

## Results

### Sample characteristics and representativeness

A total of 3794 individuals consented to participate in the study: n = 1672 were ineligible to participate due to using devices with small screens (e.g., cellphones, tablets) (n = 573) and due to oversampling of specific characteristic quota (n = 1099). Of the remaining 2122 eligible individuals, n = 540 were removed because these respondents did not complete at least one choice task. As a result, 1582 were included in the analysis set: n = 1501 completed all 16 choice tasks and n = 81 completed at least one choice task.

The sample differed statistically from the general population in all measured characteristics except for age, sex, and province/territory of residence (Table 2). Compared to the general population, the sample consisted of statistically more participants whose primary language is English, completed college education or higher, and reported poorer health based on the General Health Question; these variables were subjected to raking.

**Table 2:** Characteristics of the study population

Characteristic	Level	Number	Proportion	Population value^a^	χ^2^ statistic	P value
Gender	Male	757	0.48	0.48	0.06	0.97
	Female	819	0.52	0.52		
Age (years)	18–29	306	0.19	0.20	0.51	1.00
	30–39	272	0.17	0.17		
	40–49	329	0.21	0.21		
	50–59	285	0.18	0.18		
	60–69	184	0.11	0.11		
	70+	203	0.13	0.13		
Province or territory of residence	Alberta	171	0.11	0.12	4.65	0.98
	British Columbia	210	0.13	0.13		
	Manitoba	55	0.04	0.04		
	New Brunswick	36	0.02	0.02		
	Newfoundland and Labrador	22	0.01	0.02		
	Nova Scotia	43	0.03	0.03		
	Northwest Territory	2	0.001	0.001		
	Nunavut Territory	0	0	0.001		
	Ontario	613	0.39	0.39		
	Quebec	362	0.23	0.23		
	Prince Edward Island	6	0.004	0.004		
	Saskatchewan	58	0.04	0.04		
	Yukon Territory	1	0.001	0.001		
Primary language spoken at home	English	1228	0.78	0.58	332.0	< 0.01
	French	299	0.19	0.22		
	Other	52	0.03	0.20		
Language survey completed in	English	1301	0.82	N/A		
	French	278	0.18	N/A		
Country of birth	Canada	1263	0.84	N/A		
	Outside of canada	248	0.16			
Marital status	Single	291	0.26	0.28	85.0	< 0.01
	Legally married	702	0.47	0.48		
	In a common-law relationship	229	0.15	0.11		
	Separated, but still legally married	34	0.02	0.03		
	Divorced	100	0.07	0.06		
	Widowed	55	0.04	0.06		
Education level	No certificate, diploma or degree	40	0.03	0.15	478.1	< 0.01
	High school certificate or equivalent	302	0.20	0.24		
	Apprenticeship or trades certificate or diploma	104	0.07	0.12		
	Collage, CEGEP or other non-university certificate or diploma	372	0.25	0.20		
	University certificate or diploma below the bachelor’s level	196	0.13	0.05		
	University certificate, diploma at the bachelor’s level or above	497	0.33	0.23		
General health question	Excellent	180	0.12	0.22	27.8	< 0.01
	Very good	592	0.39	0.36		
	Good	497	0.33	0.29		
	Fair	197	0.13	0.11		
	Poor	40	0.03	0.02		

^a^Rounding of proportions to two decimal places

Respondents’ perceptions of the DCE valuation task are presented in Table 3. In general, the respondents found the presentation of the health states to be clear but found it challenging to choose between the pairs of health states on each screen. A range of choice strategies was observed amongst the respondents with considering most or all of the aspects presented to them.

**Table 3:** Respondents’ perceptions of the valuation task and their choice strategies

	Frequency	Percent
How clear was the presentation of the health states?		
Very unclear	41	2.7
Unclear	87	5.8
Neither clear nor unclear	266	17.6
Clear	792	52.4
Very clear	327	21.6
How difficult was it to choose between the pairs of health states on each screen?		
Very difficult	115	7.6
Difficult	605	40.0
Neither easy nor difficult	473	31.3
Easy	255	16.9
Very easy	65	4.3
Did you have a strategy for choosing between the pairs of health states on each screen?		
I did not have a strategy	78	5.2
I focused on just a few aspects of the health states	213	14.1
I focused on the aspects that were highlighted in yellow	356	23.5
I considered most of the aspects	431	28.5
I considered all of the aspects	408	27.0
Other	27	1.8

### Utility estimates

The conditional logit models revealed that respondents preferred additional life years (Table 4). Movements away from “no problems” (level 1) in each of the HRQL dimensions were generally valued negatively; exceptions were the positive coefficients of level 2 of the pain and levels 2 and 3 of the problems sleeping. Incremental moves to the next worst dimension level were generally associated with an absolutely larger coefficient; however, there were a few exceptions. Inconsistencies were observed between levels 2 and 3, such that level 3 was more preferred to the less severe level 2, for the following dimensions: problems sleeping, problems with support from family and/or friends, and worry that my health condition will get worse. Problems sleeping revealed an additional inconsistency for the two worst levels. Coefficients for level 2 for the pain and fatigue dimensions were negligible and therefore, combined with the level indicating no problems (level 1).

**Table 4:** Conditional logit results: Model 1 (unconstrained), Model 2 (raked and unconstrained), and Model 3 (montonicity imposed)

Mean		Model 1	Model 2	Model 3
		Coefficient^a^ (robust SE)	Coefficient^a^ (robust SE)	Coefficient^a^ (robust SE)
Duration	Linear	0.331 (0.013)***	0.345 (0.026)***	0.366 (0.023)***
Pain × Duration^a^	2	0.003 (0.007)	0.014 (0.012)	0
	3	− 0.020 (0.008)**	− 0.025 (0.018)***	− 0.021 (0.015)
	4	− 0.060 (0.008)***	− 0.062 (0.015)***	− 0.072 (0.014)***
	5	− 0.136 (0.007)***	− 0.148 (0.015)***	− 0.149 (0.014)***
Fatigue × Duration^a^	2	− 0.002 (0.007)	− 0.030 (0.013)**	− 0.020 (0.011)
	3	− 0.021 (0.006)***	− 0.038 (0.013)***	− 0.028 (0.011)**
	4	− 0.040 (0.007)***	− 0.048 (0.015)***	− 0.053 (0.014)***
	5	− 0.050 (0.007)***	− 0.069 (0.013)***	− 0.060 (0.012)***
Nausea × Duration^a^	2	− 0.032 (0.006)***	− 0.036 (0.013)***	− 0.036 (0.011)***
	3	− 0.044 (0.006)***	− 0.047 (0.011)***	− 0.055 (0.011)***
	4	− 0.061 (0.006)***	− 0.063 (0.013)***	− 0.059 (0.012)***
	5	− 0.089 (0.008)***	− 0.103 (0.015)***	− 0.109 (0.014)***
Sleep × Duration^a^	2	0.020 (0.006)***	0.024 (0.012)**	0
	3	0.015 (0.007)**	0.036 (0.015)**	0
	4	− 0.040 (0.006)***	− 0.019 (0.013)	− 0.028 (0.008)***
	5	− 0.006 (0.007)	0.010 (0.015)	− 0.028 (0.008)***
Work × Duration^a^	2	− 0.009 (0.008)	− 0.026 (0.016)	− 0.021 (0.014)
	3	− 0.014 (0.006)**	− 0.040 (0.016)**	− 0.033 (0.011)***
	4	− 0.034 (0.007)***	− 0.039 (0.013)***	− 0.033 (0.011)***
	5	− 0.076 (0.006)***	− 0.088 (0.013)***	− 0.085 (0.012)***
Support × Duration^a^	2	− 0.023 (0.007)***	− 0.034 (0.012)***	− 0.008 (0.008)
	3	− 0.001 (0.006)	0.009 (0.012)***	− 0.008 (0.008)
	4	− 0.045 (0.006)***	− 0.038 (0.013)***	− 0.044 (0.012)***
	5	− 0.067 (0.006)***	− 0.064 (0.011)***	− 0.072 (0.010)***
Sadness × Duration^a^	2	− 0.009 (0.007)	0.001 (0.012)	0
	3	− 0.035 (0.007)***	− 0.050 (0.014)***	− 0.047 (0.010)***
	4	− 0.038 (0.006)***	− 0.035 (0.013)***	− 0.047 (0.010)***
	5	− 0.058 (0.007)***	− 0.069 (0.013)***	− 0.068 (0.012)***
Worry my health will get worse × Duration^a^	2	− 0.031 (0.006)***	− 0.046 (0.011)***	− 0.035 (0.010)***
	3	− 0.019 (0.007)***	− 0.021 (0.012)	− 0.035 (0.010)***
	4	− 0.039 (0.007)***	− 0.050 (0.014)***	− 0.043 (0.011)***
	5	− 0.049 (0.007)***	− 0.033 (0.015)***	− 0.043 (0.011)***
Log-likelihood		− 15,009.31	− 14,602.54	− 14,655.46
Parameters		33	33	23
AIC		30,084.62	29,271.07	29,540.03
BIC		30,375.13	29,560.89	29,724.46

^a^The coefficient for each level of each dimension was estimated as the interaction of that level with duration

Levels of statistical significant: ***1%; **5%

*AIC* Akaike information criterion, *BIC* Bayesian information criterion

The raked weights were applied to re-estimate Model 2. The trends of utility decrements across dimension levels were generally similar between the raked and unweighted models; however, the magnitude of the decrements were observed to be larger for the raked model. More inconsistencies were observed for the sleep and sadness dimensions in the raked model versus the unweighted model.

**Fig. 1:**
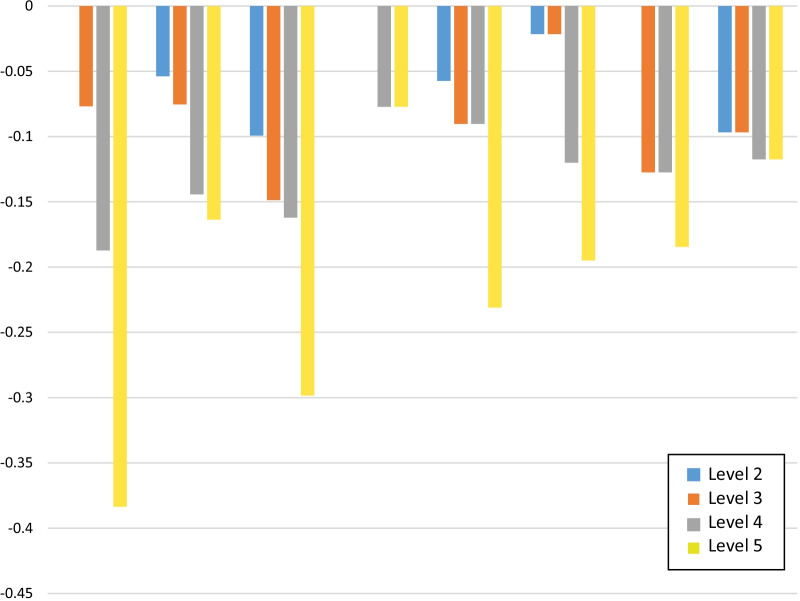
Canadian FACT-8D utility decrements by dimension and level (derived from Model 3 raked condition logit, monotonicity imposed)

Non-monotonic dimension levels in Model 2 were constrained (Model 3). The log-likelihood ratio test indicated that the unconstrained did not provide better fit that the constrained models (χ^2^ = 8.1, p = 0.3), further supporting our strategy to constrain for monotonicity within dimensions. As per the Australian FACT-8D valuation study [20], the estimates from the parsimonious Model 3 (constrained) defined the Canadian value set for the FACT-8D (Table 4). Model 3 revealed that pain, nausea, and problems with working dimensions most greatly affected the individual’s utility function; this was followed by sadness, fatigue, and worry about health condition will get worse (Fig. 1).

While the majority of respondents considered most or all of the attributes presented, attribute non-attendance may be a problem (Table 3). We conducted a post hoc enhanced latent class conditional logit model using lclogit2 and lclogitml2. The four-class model appears to be the optimal model as raising and lowering the number of classes slightly worsens BIC. The estimates of the latent class model with four latent classes is available as Additional file 1.

#### FACT-8D utility calculation

As per the conditions set by the FACIT Group, the FACT-8D is not a standalone instrument. The Canadian value set is applied to the nine items of the completed FACT-G required to obtain FACT-8D utility scores (Table 5). A utility index of one is assigned to individuals whose FACT-G responses indicate they are at level 1 of all eight dimensions of the FACT-8D (11111111): an index of − 0.65 is the worst possible state defined by the FACT-8D. For all other health states, the utility score for individual *i* is calculated as follows:

2 $$FACT - 8D_{i} = 1 - \mathop \sum \limits_{d = 1}^{8} w_{dl} |FACT - 8D_{dli} ,$$

where *w* is the utility weight for each level *l* of dimension *d* of the FACT-8D. Stata and SPSS codes for the Canadian FACT-8D value set are available as Additional file 1.

**Table 5:** FACT-8D descriptive system: how the dimensions and levels map to the 9 component FACT-G items, and associated Canadian utility decrements

FACT-8D Dimension (*d*) *FACT-G question*	FACT-G item	FACT-G item level (*l*) and associated utility decrement (*w*_*dl*_)				
		Level 1 BEST	Level 2	Level 3	Level 4	Level 5 WORST
*Pain*	GP4	Not at all	A little bit	Somewhat	Quite a bit	Very much
I have pain		0	0	− 0.077	− 0.187	− 0.384
*Fatigue (lack of energy)*	GP1	Not at all	A little bit	Somewhat	Quite a bit	Very much
I have a lack of energy		0	− 0.054	− 0.075	− 0.144	− 0.164
*Nausea*	GP2	Not at all	A little bit	Somewhat	Quite a bit	Very much
I have nausea		0	− 0.099	− 0.149	− 0.162	− 0.298
*Sleep*	GF5	Not at all	A little bit	Somewhat	Quite a bit	Very much
I am sleeping well		0	0	0	− 0.077	− 0.077
*Work*	GF1	Very much	Quite a bit	Somewhat	A little bit	Not at all
I am able to work (include work at home)		0	− 0.057	− 0.090	− 0.090	− 0.231
Support*	GS2, GS3	Very much	Quite a bit	Somewhat	A little bit	Not at all
I get emotional support from my family and support from my friends		0	− 0.022	− 0.022	− 0.120	− 0.195
*Sadness*	GE1	Not at all	A little bit	Somewhat	Quite a bit	Very much
I feel sad		0	0	− 0.127	− 0.127	− 0.185
*Worry my health will get worse*	GE6	Not at all	A little bit	Somewhat	Quite a bit	Very much
I worry that my condition will get worse		0	− 0.097	− 0.097	− 0.097	− 0.118

^*^For the Support dimension, take the better of the two items

## Discussion

Building on the work previously reported in the FACT-8D health state classification system development and Australian valuation study [20], we determined the Canadian value set for the FACT-8D, a cancer-specific algorithm for calculating utilities. The Canadian FACT-8D value set provides another decision-making tool to inform CUA in Canada, especially in situations where only the FACT-G was administered to assess patients’ HRQL. By using select item responses of the FACT-G, FACT-8D utilities can be generated which, in turn, can inform CUA.

The Canadian valuation results revealed that the main contributors of the general population respondents’ utility were pain, nausea, problems with working, and problems with support from family and/or friends; these findings were similar to those reported in the Australian valuation study [20]. Also captured in the FACT-8D classification system were dimensions reflecting other symptoms and impacts of cancer and its treatment (e.g., fatigue, sleep problems, and worry about future health). With the exception of pain and nausea, the utility decrements for the dimensions considered more cancer-sensitive were generally smaller. However, we do not anticipate this observation will reduce the impact of cancer-sensitive dimensions when the FACT-8D utilities inform CUA as other factors will affect the prevalence of the cancer-sensitive dimensions and the difference in symptom prevalence between trial arms or other comparator groups. The extent to which the inclusion of cancer-sensitive dimensions provides a more relevant and sensitive cancer-specific utility measure will depend upon the clinical context.

The Canadian general population’s valuation of the health states defined by the FACT-8D was generally monotonic within each dimension, such that poorer HRQL levels had larger utility decrements. However, when comparing the Canadian valuation results with those of Australia, both countries’ value sets revealed some slightly inconsistent orderings of utility decrements across the levels of most dimensions. Only nausea and one other dimension demonstrated no inconsistency: fatigue for Canada and pain for Australia. Sleep was problematic in both countries’ valuations. To overcome the inconsistency for this dimension, the five levels were collapsed down to two levels to capture minor and severe sleep issues. Collapsing of levels to reflect minor and severe issues was also observed for the sadness and worry dimensions. For the remaining dimensions describing pain, problems with work, problems with support with family and friends, inconsistencies were observed in the less severe levels.

The worst possible state (i.e., PITS state) defined by Canadian FACT-8D algorithm is − 0.65, which is similar in magnitude to the Australian PITS state (− 0.54). However, when compared to other MAUIs, the Canadian FACT-8D PITS state is significantly lower than that of the EQ-5D-5L (− 0.15) [30] and the cancer-specific utility instrument, QLU-C10D (− 0.15) [12]. While the difference between the PITS values of the FACT-8D and the EQ-5D-5L may be result of different valuation methods used (e.g., FACT-8D used the DCE whereas EQ-5D-5L used TTO), comparing the PITS values for the FACT-8D and the QLU-C10D offers a more informative comparison. Assessing the common dimensions between the two cancer-specific algorithms reveals that most severe levels of pain, fatigue, and nausea demonstrates greater disutilities for the FACT-8D. This may be a result of the extra response level in the FACT-8D but this will need to be explored in the future.

The conditional logit was selected over the mixed logit results because economic evaluation is mostly concerned with the mean response; preference heterogeneity is a secondary concern. The choice of a monotonic main-effects model for calculating utility is readily accessible for a range of end users, clinical interpretable and consistent with the FACIT quality of life conceptual model.

The FACT-8D health state classification was valued using a DCE. This approach has a strong theoretical measurement framework, well established statistically robust experimental design and modelling methods, and demonstrated feasibility with online recruitment and data collection [29, 31, 32]. In this study, respondents appraised choice sets containing nine attributes. The relatively larger number of attributes raised concerns regarding the cognitive burden of the respondents; however, we conducted innovative work to have only four attributes differ in each choice set. Although previous work revealed that the respondents preferred format of yellow highlighting [22], the concerns of respondents employing heuristics, such as considering a single attribute, to trade-off between other attributes, were alleviated when the majority of the respondents considered most or all of the attributes presented to them.

There are limitations associated with this study. While the valuation survey sample consisted of a large number of respondents with quota sample sampling achieving population representativeness for age, sex, and province/territory of residence, the study sample tended to have higher education and report poorer health. To overcome this limitation, we raked the study sample on under represented characteristics.

The influence of sociodemographic variables to the utility estimates will be assessed in future analysis of pooled data from international valuations of the FACT-8D. While it may seem like having general population respondents valuing these health states is a limitation due to their inexperience with cancer, it is important to adopt an extra-welfarist approach as a publicly-funded healthcare system exists in Canada. As such, the preferences from general population respondents should maximize societal health. The health state descriptions make no mention of “cancer”, alleviating any potential stereotypes respondents may have in regards to a cancer diagnosis; although previous research has revealed that disease labels do not affect health state valuations [33]. Further, due to a logistical oversight during survey implementation, individuals on devices with small screen sizes (e.g., cellphones, tablets) were initially allowed into the survey but then encountered a system initiated timeout that prevent them from proceeding to the DCE component; as such, the reported rate of participant involvement is not accurate due to the device used.

We used the FACT-8D valuation methods developed by King et al. [20]. This involved reversing the direction of the phrasing for the three positively worded FACT-G dimensions (i.e., work, sleep, and support), a solution that solved initial problems in the patterns of utility decrements in the developmental work conducted in Australia [20]. While reframing the positively worded dimensions as problem statements in the DCE reduced the cognitive burden placed on the respondents when completing the choice tasks, this posed the question of how to map the corresponding positively framed FACT-G responses to utility decrements in the FACT-8D utility scoring algorithm. The members of the MAUCa Consortium propose mapping to the original FACT-G item wording by reverse scoring when calculating utility decrements, as shown in Table 1 and the scoring instructions in the Additional file 1. We acknowledge that when used as self-reported health items, negatively and positively framed versions of the work, sleep, and support items would not necessarily yield mirror image results. However, this solution is pragmatic in allowing utility values to be generated from existing FACT-G datasets, with the reframing used solely for the purpose of making the DCE valuation task more feasible for participants. Better solutions may be found in future research, particularly if similar problems arise in other valuation DCEs with positively and negatively framed items. The measurement properties of the FACT-8D (using the Australian value set) has been tested against the EQ-5D-5L (scored using the UK 3L crosswalk and the 5L England value set) [34]. The FACT-8D demonstrated good convergent validity and responsiveness but the EQ-5D-5L showed better known groups’ validity.

We did not test potential interactions between pairs of FACT-8D dimensions although we acknowledge that they may exist. The influence of potential interactions could be explored in the future both quantitatively and qualitatively. We opted for a more parsimonious approach as testing all possible interactions would require an unfeasibly large sample size due to the many additional coefficients that would need to be estimated from the DCE data. The model presented in the article excluded complex interactions and therefore, is clinically interpretable and make it comprehensible to end-users.

In our work, we anchored the utilities on the standard QALY scale using a common approach [29]. The resulting Canadian FACT-8D value set assumes the zero condition (i.e., a health state of zero duration is equivalent to the dead state). However, previous work has revealed that different DCE-based approaches to anchor utility scores can have varying impact on the generated utilities [35]. This may be a limitation when value sets determined by DCEs are used to guide resource allocation decisions. While it is possible to anchor utilities by including dead as a health state within the DCE, this is problematic within a random utility theory framework as some respondents may never acknowledge a health state to be less preferred than immediate death [36].

Results from the study add to the continuing debate amongst health economists regarding the use of generic versus cancer-specific utility instruments in informing resource allocation decisions. The FACT-8D contains a large number of dimensions specific to cancer. While the FACT-G dimensions are more sensitive in capturing cancer patients’ HRQL, the resulting metrics make it difficult to compare to CUA results in other therapeutic areas. Future work is needed on accessing the acceptability of cancer-specific utility instruments in informing resource allocation decisions.

## Conclusions

The largest impacts on utility included three generic dimensions (i.e., pain, support, and work) and nausea, a symptom caused by cancer (e.g., brain tumours, gastrointestinal tumours, malignant bowel obstruction) and by common treatments (e.g., chemotherapy, radiotherapy, opioid analgesics). Our findings demonstrate that cancer-specific utilities can be determined using responses to the FACT-G (as well as many FACIT measures that embed the FACT-G items); this, in turn, facilitates CUA for cancer interventions from a Canadian perspective. The widespread use of the FACT-G to measure quality of life outcomes of cancer patients will enable utilities not only to be estimated prospectively but also from a large number of retrospective studies. While the results reveal that the Canadian value set for the FACT-8D is similar to the Australian value set [20], CADTH recommends that preferences of the Canadian general population should be the reference case to guide societal decisions [1]. The Canadian FACT-8D value set affords cancer-specific utility weights that may be more sensitive to differences resulting from cancer care than a generic MAUI, which may be more informative in guiding cancer priority setting and resource allocation decisions in Canada. We intend to conduct head-to-head comparisons of the FACT-8D versus generic MAUIs assess its performance. The availability of more QALY estimates and CUAs will enable decision makers to be more informed when allocating resources in Canada’s publicly funded health care system.

## Supplementary Information

**Additional file 1:** Supplementary Appendix and Table.

## Data Availability

The data that support the findings of this study are available on request from the corresponding author.

## Abbreviations

CUA: Cost-utility analysis
DCE: Discrete choice experiment
EORTC: European Organization of Research and Treatment in Cancer
FACT-G: Functional Assessment of Cancer Therapies-General version
FACT-GP: Functional Assessment of Cancer Therapies-General population version
HRQL: Health-related quality of life
HUI: Health Utility Index
MAUCa: Multi-attribute utility in cancer
MAUI: Multi-attribute utility instrument
QALY: Quality-adjusted life years
