# Supplementary material for: The FACT-8D, a new cancer-specific utility algorithm based on the Functional Assessment of Cancer Therapies-General (FACT-G): a Canadian valuation study
Source: Health Qual Life Outcomes. 2022 Jun 16;20:97. doi: 10.1186/s12955-022-02002-z (PMC9205108; doi:10.1186/s12955-022-02002-z)
Supplement: Supplementary file 1 — Additional file 1: Supplementary Appendix and Table. [file 12955_2022_2002_MOESM1_ESM.docx]

**The FACT-8D, a new cancer-specific utility algorithm based on the Functional Assessment of Cancer Therapies-General (FACT-G): a Canadian valuation study**

BMC Health and Quality of Life Outcomes

Helen McTaggart-Cowan (hcowan@bccrc.ca), Madeleine T. King, Richard Norman, Daniel S.J. Costa, A. Simon Pickard, Rosalie Viney, Stuart Peacock

**Supplementary Appendix: FACT-8D utility algorithm and scoring instructions**

This appendix contains instructions for calculating FACT-8D utility scores from FACT-G responses, whether collected from the FACT-G or any related FACIT instruments containing FACT-G items.

**Instructions**

For any individual *i* who has provided responses to the nine FACT-G items in Table 1, the individual’s FACT-8D utility score is calculated as follows.

First, determine the corresponding level *l* for each dimension *d* and the associated utility decrement

(*w­_dl_*), following the mapping of FACT-G item levels to Australian FACT-8D utility decrements in Table 1 (estimated from the Canadian general population).

**FACT-8D scoring algorithm**

A utility score of 1 is assigned to individuals who FACT-G scores indicate they are at level 1 of all 8 dimensions of the FACT-8D. For all other health states, the utility score is 1 minus each the utility weight (*w­_dl_*) (hence a net utility decrement) for each level down from no problems in each of the 8 FACT-8D dimensions.

$${FACT\text{-}8D}_{i}=1-\sum_{d=1}^{8} w_{dl}|{FACT\text{-}8D}_{dli}$$

For example, a health state with *quite a bit of pain*, *somewhat lacking energy*, *not at all able to work*, *feeling a little bit sad*, *getting very much emotional support from family and friend*s, *sleeping very (much) well*, *no nausea*, and *not at all worried that condition will worse*, would be valued at 1 minus the decrements for *Pain* level 4, *Fatigue* level 3, and *Work* level 5 = 1 – 0.187 – 0.075 – 0.231 = 0.507. The best possible health state has a value of 1, and the worst possible state has a value of -0.65 (1 – 0.384 – 0.164 – 0.298 – 0.077 – 0.231 – 0.195 – 0.185 – 0.117).

**STATA code to calculate FACT-8D utility scores from FACT-G responses using Canadian value set**

Written by Richard Norman [richard.norman@curtin.edu.au](mailto:richard.norman@curtin.edu.au) and adapted by Helen McTaggart-Cowan hcowan@bccrc.ca

22 November 2021

*This code is designed to convert FACT-G responses into FACT-8D utility weights. It uses the Canadian

*DCE-derived weights developed by Helen McTaggart-Cowan.

*It is based on the assumption that the underlying data are coded between 1 and 5, where 1 means

*'Not at all', 2 means 'A little bit', 3 means 'Somewhat', 4 means 'Quite a bit', and 5 means 'Very much'. *The coding of the variables is clustered by dimension, so Physical Well-Being items are labelled GP1-*GP7, Social / Family Well-Being items are labelled GS1-GS7, Emotional Well-Being items are labelled *GE1-GE6, and Functional Well-Being are labelled GF1-GF7.

gen pai = gp4

gen fat = gp1

gen nau = gp2

gen sle = 4-gf5

gen wrk = 4-gf1

gen sup = 4 - max(gs2,gs3)

gen sad = ge1

gen wor = ge6

gen paidec=.

replace paidec=0 if pai==1

replace paidec=0 if pai==2

replace paidec=-0.077 if pai==3

replace paidec=-0.187 if pai==4

replace paidec=-0.384 if pai==5

gen fatdec=.

replace fatdec=0 if fat==1

replace fatdec=0 if fat==2

replace fatdec=-0.054 if fat==3

replace fatdec=-0.144 if fat==4

replace fatdec=-0.164 if fat==5

gen naudec=.

replace naudec=0 if nau==1

replace naudec=-0.099 if nau==2

replace naudec=-0.149 if nau==3

replace naudec=-0.162 if nau==4

replace naudec=-0.298 if nau==5

gen sledec=.

replace sledec=0 if sle==1

replace sledec=0 if sle==2

replace sledec=0 if sle==3

replace sledec=-0.077 if sle==4

replace sledec=-0.077 if sle==5

gen wrkdec=.

replace wrkdec=0 if wrk==1

replace wrkdec=-0.057 if wrk==2

replace wrkdec=-0.090 if wrk==3

replace wrkdec=-0.090 if wrk==4

replace wrkdec=-0.231 if wrk==5

gen supdec=.

replace supdec=0 if sup==1

replace supdec=-0.022 if sup==2

replace supdec=-0.022 if sup==3

replace supdec=-0.120 if sup==4

replace supdec=-0.195 if sup==5

gen saddec=.

replace saddec=0 if sad==1

replace saddec=0 if sad==2

replace saddec=-0.127 if sad==3

replace saddec=-0.127 if sad==4

replace saddec=-0.185 if sad==5

gen wordec=.

replace wordec=0 if wor==1

replace wordec=-0.097 if wor==2

replace wordec=-0.097 if wor==3

replace wordec=-0.097 if wor==4

replace wordec=-0.117 if wor==5

gen fact8d = 1 + paidec + fatdec + naudec + sledec + wrkdec + supdec + saddec + wordec

**SPSS code to calculate FACT-8D utility scores from FACT-G responses using Canadian value set**

Written by Daniel Costa [daniel.costa@sydney.edu.au](mailto:daniel.costa@sydney.edu.au) and adapted by Helen McTaggart-Cowan [hcowan@bccrc.ca](mailto:hcowan@bccrc.ca)

22 November 2021

* Encoding: UTF-8.

* Encoding: .

* This code is designed to convert FACT-G responses into FACT-8D utility weights. It uses the Canadian

* DCE-derived weights developed by Helen McTaggart-Cowan. It is based on the assumption that the

* underlying data are coded between 1 and 5, where 1 means 'Not at all', 2 means 'A little bit', 3 means * 'Somewhat', 4 means 'Quite a bit', and 5 means 'Very much'. The coding of the variables is clustered by * domain, so Physical Well-Being items are labelled GP1-GP7, Social / Family Well-Being items are

* labelled GS1-GS7, Emotional Well-Being items are labelled GE1-GE6, and Functional Well-Being are

* labelled GF1-GF7.

compute pai = gp4.

compute fat = gp1.

compute nau = gp2.

compute sle = 4-gf5.

compute wrk = 4-gf1.

compute sup = 4 - max(gs2,gs3).

compute sad = ge1.

compute wor = ge6.

exe.

compute paidec=$sysmis.

if pai=1 paidec=0.

if pai=2 paidec=0.

if pai=3 paidec=-0.077.

if pai=4 paidec=-0.187.

if pai=5 paidec=-0.384.

compute fatdec= $sysmis.

if fat=1 fatdec=0.

if fat=2 fatdec=-0.054.

if fat=3 fatdec=-0.075.

if fat=4 fatdec=-0.144.

if fat=5 fatdec=-0.164.

compute naudec=$sysmis.

if nau=1 naudec=0.

if nau=2 naudec=-0.099.

if nau=3 naudec=-0.149.

if nau=4 naudec=-0.162.

if nau=5 naudec=-0.298.

compute sledec=$sysmis.

if sle=1 sledec=0.

if sle=2 sledec=0.

if sle=3 sledec=0.

if sle=4 sledec=-0.077.

if sle=5 sledec=-0.077.

compute wrkdec=$sysmis.

if wrk=1 wrkdec=0.

if wrk=2 wrkdec=-0.057.

if wrk=3 wrkdec=-0.090.

if wrk=4 wrkdec=-0.090.

if wrk=5 wrkdec=-0.231.

compute supdec=$sysmis.

if sup=1 supdec=0.

if sup=2 supdec=-0.022.

if sup=3 supdec=-0.022.

if sup=4 supdec=-0.120.

if sup=5 supdec=-0.195.

compute saddec=$sysmis.

if sad=1 saddec=0.

if sad=2 saddec=0.

if sad=3 saddec=-0.127.

if sad=4 saddec=-0.127.

if sad=5 saddec=-0.185.

compute wordec=$sysmis.

if wor=1 wordec=0.

if wor=2 wordec=-0.097.

if wor=3 wordec=-0.097.

if wor=4 wordec=-0.097.

if wor=5 wordec=-0.117.

compute fact8d = 1 + paidec + fatdec + naudec + sledec + wrkdec + supdec + saddec + wordec.

exe.

**Table S1: Latent class model results**

| **Class** | **Dimension** | **Level** | **Coefficient** | **Standard error** | **p-value** | **95% Confidence interval** |
| --- | --- | --- | --- | --- | --- | --- |
| 1  (Class share: 0.89) | Duration | Linear | 2.878 | 0.185 | 0 | 2.515, 3.240 |
|  | Pain x Duration | 2 | -0.219 | 0.049 | 0 | -0.315, -0.123 |
|  |  | 3 | -0.346 | 0.100 | 0.001 | -0.542, -0.150 |
|  |  | 4 | -0.492 | 0.073 | 0 | -0.636, -0.348 |
|  |  | 5 | -0.883 | 0.086 | 0 | -1.052, -0.714 |
|  | Fatigue x Duration | 2 | -0.688 | 0.844 | 0 | -0.854, -0.523 |
|  |  | 3 | -0.334 | 0.063 | 0 | -0.458, -0.209 |
|  |  | 4 | -0.615 | 0.083 | 0 | -0.778, -0.453 |
|  |  | 5 | -0.928 | 0.088 | 0 | -1.101, -0.756 |
|  | Nausea x Duration | 2 | -0.159 | 0.058 | 0.006 | -0.273, -0.046 |
|  |  | 3 | 0.103 | 0.055 | 0.060 | -0.004, 0.211 |
|  |  | 4 | -0.427 | 0.053 | 0.424 | -0.147, 0.062 |
|  |  | 5 | -0.427 | 0.063 | 0 | -0.551, -0.303 |
|  | Sleep x Duration | 2 | 0.219 | 0.058 | 0 | 0.106, 0.332 |
|  |  | 3 | 0.155 | 0.073 | 0.035 | 0.011, 0.299 |
|  |  | 4 | -0.002 | 0.060 | 0.976 | -0.119, 0.115 |
|  |  | 5 | 0.226 | 0.047 | 0 | 0.134, 0.317 |
|  | Work x Duration | 2 | 0.176 | 0.092 | 0.06 | -0.004, 0.357 |
|  |  | 3 | 0.160 | 0.047 | 0.001 | 0.068, 0.253 |
|  |  | 4 | 0.265 | 0.063 | 0 | 0.142, 0.388 |
|  |  | 5 | -0.372 | 0.049 | 0 | -0.469, -0.275 |
|  | Support x Duration | 2 | -0.007 | 0.067 | 0.918 | -0.139, 0.125 |
|  |  | 3 | 0.329 | 0.074 | 0 | 0.185, 0.473 |
|  |  | 4 | -0.796 | 0.069 | 0 | -0.932, -0.660 |
|  |  | 5 | -0.504 | 0.058 | 0 | -0.617, -0.390 |
|  | Sadness x Duration | 2 | 0.449 | 0.068 | 0 | 0.315, 0.582 |
|  |  | 3 | -0.295 | 0.0650 | 0 | -0.422, -0.167 |
|  |  | 4 | -0.007 | 0.070 | 0.916 | -0.145, 0.130 |
|  |  | 5 | -0.234 | 0.057 | 0 | -0.346, -0.122 |
|  | Worry that my health will get worse x Duration | 2 | -0.254 | 0.064 | 0 | -0.379, -0.129 |
|  |  | 3 | 0.026 | 0.089 | 0.766 | -0.147, 0.200 |
|  |  | 4 | -0.270 | 0.052 | 0 | -0.372, -0.169 |
|  |  | 5 | -0.367 | 0.049 | 0 | -0.464, -0.271 |
| 2  (Class share: 0.311) | Duration | Linear | 0.157 | 0.019 | 0 | 0.119, 0.195 |
|  | Pain x Duration | 2 | 0.016 | 0.010 | 0.124 | -0.004, 0.036 |
|  |  | 3 | -0.011 | 0.013 | 0.410 | -0.037, 0.015 |
|  |  | 4 | -0.016 | 0.013 | 0.227 | -0.041, 0.010 |
|  |  | 5 | -0.058 | 0.011 | 0 | -0.803, -0.036 |
|  | Fatigue x Duration | 2 | 0.005 | 0.011 | 0.653 | -0.170, 0.028 |
|  |  | 3 | -0.011 | 0.013 | 0.410 | -0.037, 0.015 |
|  |  | 4 | -0.015 | 0.012 | 0.207 | -0.039, 0.008 |
|  |  | 5 | -0.029 | 0.010 | 0.005 | -0.049, -0.009 |
|  | Nausea x Duration | 2 | -0.011 | 0.010 | 0.291 | -0.31, 0.009 |
|  |  | 3 | -0.021 | 0.010 | 0.030 | -0.040, -0.002 |
|  |  | 4 | -0.017 | 0.010 | 0.089 | -0.036, 0.003 |
|  |  | 5 | -0.031 | 0.012 | 0.009 | -0.054, -0.008 |
|  | Sleep x Duration | 2 | 0.024 | 0.009 | 0.009 | 0.006, 0.042 |
|  |  | 3 | 0.008 | 0.011 | 0.451 | -0.013, 0.030 |
|  |  | 4 | -0.023 | 0.011 | 0.038 | -0.044, -0.001 |
|  |  | 5 | -0.0060 | 0.011 | 0.594 | -0.028, 0.016 |
|  | Work x Duration | 2 | 0.014 | 0.012 | 0.240 | -0.009, 0.036 |
|  |  | 3 | 0.004 | 0.011 | 0.708 | -0.174, 0.026 |
|  |  | 4 | -0.017 | 0.010 | 0.100 | -0.036, 0.003 |
|  |  | 5 | -0.042 | 0.009 | 0 | -0.061, -0.024 |
|  | Support x Duration | 2 | 0.008 | 0.011 | 0.465 | -0.013, 0.029 |
|  |  | 3 | 0.008 | 0.009 | 0.404 | -0.010, 0.026 |
|  |  | 4 | -0.015 | 0.010 | 0.128 | -0.035, 0.004 |
|  |  | 5 | -0.037 | 0.010 | 0 | -0.056, -0.017 |
|  | Sadness x Duration | 2 | -0.006 | 0.010 | 0.531 | -0.027, 0.014 |
|  |  | 3 | -0.020 | 0.012 | 0.078 | -0.043, 0.002 |
|  |  | 4 | -0.018 | 0.011 | 0.085 | -0.039, 0.003 |
|  |  | 5 | -0.037 | 0.012 | 0.002 | -0.061, -0.013 |
|  | Worry that my health will get worse x Duration | 2 | -0.021 | 0.010 | 0.036 | -0.041, -0.001 |
|  |  | 3 | -0.019 | 0.011 | 0.095 | -0.041, 0.003 |
|  |  | 4 | -0.021 | 0.012 | 0.075 | -0.044, 0.002 |
|  |  | 5 | -0.036 | 0.011 | 0.001 | -0.057, -0.015 |
| 3  (Class share: 0.171) | Duration | Linear | 0.385 | 0.090 | 0 | 0.208, 0.561 |
|  | Pain x Duration | 2 | -0.020 | 0.036 | 0.572 | -0.090, 0.500 |
|  |  | 3 | -0.124 | 0.088 | 0.157 | -0.297, 0.048 |
|  |  | 4 | -0.229 | 0.087 | 0.009 | -0.400, -0.058 |
|  |  | 5 | -0.304 | 0.074 | 0 | -0.449, -0.160 |
|  | Fatigue x Duration | 2 | -0.089 | 0.041 | 0.032 | -0.170, -0.008 |
|  |  | 3 | -0.039 | 0.058 | 0.506 | -0.153, 0.076 |
|  |  | 4 | -0.020 | 0.049 | 0.681 | -0.117, 0.077 |
|  |  | 5 | -0.172 | 0.040 | 0 | -0.250, -0.093 |
|  | Nausea x Duration | 2 | -0.090 | 0.054 | 0.096 | -0.197, 0.016 |
|  |  | 3 | -0.202 | 0.075 | 0.007 | -0.350, -0.055 |
|  |  | 4 | -0.162 | 0.054 | 0.003 | -0.268, -0.057 |
|  |  | 5 | -0.289 | 0.072 | 0 | -0.430, -0.148 |
|  | Sleep x Duration | 2 | -0.001 | 0.031 | 0.968 | -0.061, 0.059 |
|  |  | 3 | 0.009 | 0.0136 | 0.806 | -0.062, 0.080 |
|  |  | 4 | -0.093 | 0.037 | 0.013 | -0.166, -0.020 |
|  |  | 5 | -0.098 | 0.060 | 0.100 | -0.215, 0.019 |
|  | Work x Duration | 2 | -0.078 | 0.062 | 0.209 | -0.199, 0.043 |
|  |  | 3 | -0.049 | 0.034 | 0.153 | -0.116, 0.018 |
|  |  | 4 | -0.120 | 0.038 | 0.001 | -0.194, -0.046 |
|  |  | 5 | -0.170 | 0.040 | 0 | -0.249, -0.091 |
|  | Support x Duration | 2 | -0.116 | 0.083 | 0.162 | -0.279, 0.047 |
|  |  | 3 | -0.005 | 0.038 | 0.893 | -0.080, 0.069 |
|  |  | 4 | -0.067 | 0.045 | 0.136 | -0.156, 0.021 |
|  |  | 5 | -0.201 | 0.037 | 0 | -0.274, -0.127 |
|  | Sadness x Duration | 2 | -0.048 | 0.059 | 0.414 | -0.162, 0.067 |
|  |  | 3 | -0.137 | 0.056 | 0.014 | -0.246, -0.028 |
|  |  | 4 | -0.156 | 0.041 | 0 | -0.236, -0.076 |
|  |  | 5 | -0.218 | 0.048 | 0 | -0.313, -0.124 |
|  | Worry that my health will get worse x Duration | 2 | -0.084 | 0.068 | 0.214 | -0.217, 0.049 |
|  |  | 3 | 0.038 | 0.045 | 0.406 | -0.051, 0.127 |
|  |  | 4 | -0.132 | 0.080 | 0.098 | -0.288, 0.024 |
|  |  | 5 | -0.018 | 0.030 | 0.554 | -0.076, 0.041 |
| 4  (Class share: 0.429) | Duration | Linear | 1.712 | 0.328 | 0 | 1.070 |
|  | Pain x Duration | 2 | 0.081 | 0.042 | 0.053 | -0.001. 0.163 |
|  |  | 3 | -0.294 | 0.065 | 0 | -0.422. -0.167 |
|  |  | 4 | -0.573 | 0.126 | 0 | -0.821, -0.325 |
|  |  | 5 | -0.887 | 0.122 | 0 | -1.125, -0.648 |
|  | Fatigue x Duration | 2 | 0.043 | 0.045 | 0.347 | -0.047, 0.133 |
|  |  | 3 | -0.225 | 0.053 | 0 | -0.328, -0.121 |
|  |  | 4 | -0.112 | 0.048 | 0.020 | -0207, -0.018 |
|  |  | 5 | -0.234 | 0.069 | 0.001 | -0.369, -0.099 |
|  | Nausea x Duration | 2 | -0.144 | 0.063 | 0.023 | -0.268, -0.020 |
|  |  | 3 | -0.280 | 0.063 | 0 | -0.405, -0.156 |
|  |  | 4 | -0.249 | 0.053 | 0 | -0.353, -0.146 |
|  |  | 5 | -0.252 | 0.076 | 0.001 | -0.402, -0.103 |
|  | Sleep x Duration | 2 | 0.272 | 0.057 | 0 | 0.161, 0.383 |
|  |  | 3 | 0.182 | 0.062 | 0.003 | 0.061, 0.303 |
|  |  | 4 | -0.286 | 0.051 | 0 | -0.386, -0.186 |
|  |  | 5 | -0.066 | 0.044 | 0.129 | -0.152, 0.019 |
|  | Work x Duration | 2 | -0.007 | 0.064 | 0.918 | -0.132, 0.119 |
|  |  | 3 | -0.326 | 0.068 | 0 | -0.460, -0.193 |
|  |  | 4 | -0.169 | 0.080 | 0.035 | -0.326, -0.012 |
|  |  | 5 | -0.296 | 0.089 | 0 | -0.570, -0.221 |
|  | Support x Duration | 2 | -0.063 | 0.035 | 0.074 | -0.133, 0.006 |
|  |  | 3 | 0.089 | 0.033 | 0.007 | 0.024, 0.154 |
|  |  | 4 | -0.241 | 0.083 | 0.04 | -0.405, -0.078 |
|  |  | 5 | -0.269 | 0.066 | 0 | -0.398, -0.140 |
|  | Sadness x Duration | 2 | -0.097 | 0.070 | 0.166 | -0.235, 0.40 |
|  |  | 3 | -0.083 | 0.049 | 0.091 | -0.178, 0.013 |
|  |  | 4 | -0.463 | 0.097 | 0 | -0.654, -0.272 |
|  |  | 5 | -0.560 | 0.105 | 0 | -0.765, -0.355 |
|  | Worry that my health will get worse x Duration | 2 | -0.168 | 0.039 | 0 | -0.245, -0.091 |
|  |  | 3 | -0.171 | 0.046 | 0 | -0.261, -0.081 |
|  |  | 4 | -0.191 | 0.070 | 0.007 | -0.329, -0.053 |
|  |  | 5 | -0.261 | 0.047 | 0 | -0.0352, -0.169 |
